# Antibody against Arenaviruses in Humans, Southwestern United States

**DOI:** 10.3201/eid2009.140593

**Published:** 2014-09

**Authors:** Mary L. Milazzo, Jon Iralu, Charles F. Fulhorst, Frederick Koster

**Affiliations:** University of Texas Medical Branch, Galveston, Texas, USA (M.L. Milazzo, C.F. Fulhorst);; Gallup Indian Medical Center, Gallup, New Mexico, USA (J. Iralu);; University of New Mexico, Albuquerque, New Mexico, USA (F. Koster)

**Keywords:** arenaviruses, viruses, antibodies, humans, zoonoses, vector-borne infections, Arizona, New Mexico, southwestern United States

**To The Editor:** Woodrats (*Neotoma* spp.) are natural hosts of Whitewater Arroyo virus (WWAV) and other Tacaribe serocomplex viruses (family *Arenaviridae*) in the western United States and northern Mexico (*1*). The results of a previous study (*2*) suggested that WWAV or Tacaribe serocomplex viruses antigenically closely related to WWAV are etiologic agents of severe febrile illnesses in humans in the United States. We note that Junín virus and other South American Tacaribe serocomplex viruses are etiologic agents of hemorrhagic fever in humans (*3*).

To further our knowledge of the epidemiology of the North American Tacaribe serocomplex viruses, we tested serum samples from hospitalized persons in a study of thrombocytopenic febrile illnesses that mimicked hantavirus pulmonary syndrome for IgG against arenaviruses. The 173 study participants were hospitalized during 1993–2001 in Arizona and New Mexico, United States. The study protocol was approved by the University of New Mexico Human Research Review Committee and the Navajo Nation Institutional Review Board. Ages of the study participants ranged from 9 to 86 years (mean 40 years). Virtually all serum samples were acute-phase specimens, and a specific diagnosis was achieved for only 55 (31.8%) of the 173 study participants.

Serum samples were tested for IgG against WWAV, Amaparí virus (AMAV), an arenavirus that is antigenically closely related to the Tacaribe serocomplex viruses known to cause hemorrhagic fever (*4*), and lymphocytic choriomeningitis virus (LCMV), the prototypical arenavirus and member of the Lassa–lymphocytic choriomeningitis serocomplex, by using an ELISA. (*5*). Briefly, we tested serial 4-fold dilutions (1:80–1:5,120) of each sample and compared results with results for negative control antigens. The adjusted optical density (AOD) of a sample-antigen reaction was the OD associated with the test antigen minus the OD associated with the corresponding control antigen. A sample was considered positive if the AOD at 1:80 was ≥0.250, the AOD at 1:320 was ≥0.250, and the sum of the AOD for the series of 4-fold dilutions was ≥0.750. The criteria for positivity were based on results of ELISA for serum samples from febrile persons who did not participate in this study and were negative for IgG against WWAV, AMAV, and LCMV.

The IgG titer against a test antigen in a positive sample was the reciprocal of the highest dilution for which the AOD was ≥0.250. Titers <320 were considered to be 160 in comparisons of titers for WWAV, AMAV, and LCMV in individual samples. The apparent homologous virus in a positive sample was the virus associated with the highest titer if the absolute value of the differences between the highest titer and titers for the 2 other viruses were ≥4-fold.

IgG against WWAV was found in acute-phase samples from 8 (4.6%) of the 173 study participants. None of the 173 study participants were positive for IgG against AMAV or LCMV. The IgG titers against WWAV in the positive samples were 320 (n = 1), 1,280 (n = 3), and ≥5,120 (n = 4). WWAV was the apparent homologous virus in the 7 persons with antibody titers ≥1,280. The apparent homologous virus in the person with the titer of 320 could not be determined from ELISA data. The presence of IgG against WWAV in acute-phase serum samples (all collected within 10 days of illness onset) implied past infection with WWAV or an arenavirus antigenically closely related to WWAV.

The state of residence (2 from Arizona, 6 from New Mexico), sex ratio (4 male patients:4 female patients), and mean age (36 years, range 16–47 years) of antibody-positive persons reflected the characteristics of the entire study population. The clinical features in each of the antibody-positive persons included fever, headache, myalgia, and thrombocytopenia. The diagnoses given for these persons were acute parvovirus infection (n = 1) by IgM assay, adult respiratory distress syndrome (n = 1) by clinical progression, and not determined (n = 6).

The results of this study indicate that a small fraction of the adult population in the southwestern United States has been infected with North American Tacaribe serocomplex virus(es). We note that the dominant epitopes in ELISA for IgG against arenaviruses are associated with the viral nucleocapsid (N) protein, and that amino acid sequence of the N protein of WWAV and amino acid sequences of N proteins of other Tacaribe arenaviruses from Arizona or New Mexico showed differences as high as 15.1% in a previous study (*1*).

It might be the case that human IgG against some Tacaribe serocomplex viruses in the southwestern United States does not react strongly against WWAV in ELISA. If so, the true prevalence of antibody against North American Tacaribe serocomplex viruses in this study might be >4.6%. Accordingly, future work should include development of broadly reactive assays for detection of human IgM and human IgG against North American Tacaribe serocomplex viruses, including those associated with wild rodents in Mexico (*6**,**7*).
